# The Usefulness of Clinical-Practice-Based Laboratory Data in Facilitating the Diagnosis of Dengue Illness

**DOI:** 10.1155/2013/198797

**Published:** 2013-12-19

**Authors:** Jien-Wei Liu, Ing-Kit Lee, Lin Wang, Rong-Fu Chen, Kuender D. Yang

**Affiliations:** ^1^Division of Infectious Diseases, Department of Internal Medicine, Kaohsiung Chang Gung Memorial Hospital, Kaohsiung 833, Taiwan; ^2^Infection Control Team, Kaohsiung Chang Gung Memorial Hospital, Kaohsiung 833, Taiwan; ^3^Chang Gung University College of Medicine, Tao-Yuan 333, Taiwan; ^4^Department of Pediatrics, Kaohsiung Chang Gung Memorial Hospital, Kaohsiung 833, Taiwan; ^5^Department of Medical Research, Show Chwan Memorial Hospital-Chang Bing, Changhua 500, Taiwan

## Abstract

Alertness to dengue and making a timely diagnosis is extremely important in the treatment of dengue and containment of dengue epidemics. We evaluated the complementary role of clinical-practice-based laboratory data in facilitating suspicion/diagnosis of dengue. One hundred overall dengue (57 dengue fever [DF] and 43 dengue hemorrhagic fever [DHF]) cases and another 100 nondengue cases (78 viral infections other than dengue, 6 bacterial sepsis, and 16 miscellaneous diseases) were analyzed. We separately compared individual laboratory variables (platelet count [PC] , prothrombin time [PT], activated partial thromboplastin time [APTT], alanine aminotransferase [ALT], and aspartate aminotransferase [AST]) and varied combined variables of DF and/or DHF cases with the corresponding ones of nondengue cases. The sensitivity, specificity, accuracy, positive predictive value (PPV), and negative predictive value (NPV) in the diagnosis of DF and/or DHF were measured based on these laboratory variables. While trade-off between sensitivity and specificity, and/or suboptimal PPV/NPV was found at measurements using these variables, prolonged APTT + normal PT + PC < 100 × 10^9^ cells/L had a favorable sensitivity, specificity, PPV, and NPV in diagnosis of DF and/or DHF. In conclusion, these data suggested that prolonged APTT + normal PT + PC < 100 × 10^9^ cells/L is useful in evaluating the likelihood of DF and/or DHF.

## 1. Introduction 

Dengue is a major medical and public health problem in tropical and subtropical regions. It is estimated that more than 2.5 billion people are living in geographic locales where dengue is endemic, and 50–100 million people have been annually infected by dengue virus (DENV) [1]. The spectrum of clinical manifestations of dengue ranges from a mild-form nonspecific febrile illness, classic dengue fever (DF), to the severe-form dengue hemorrhagic fever (DHF) [2, 3]. DHF is characterized by the presence of hemorrhagia, thrombocytopenia (<100 × 10^9^ cells/L), and clinical evidence of plasma leak resulting from increased vascular permeability [2, 3]. Based on the World Health Organization (WHO) criteria, the severity of DHF was categorized into grades I–IV as follows. DHF grade I is manifested by fever accompanied by nonspecific constitutional symptoms, with a positive tourniquet test result; DHF grade II is the appearance of spontaneous bleeding in addition to constitutional symptoms; DHF grade III is circulatory failure with signs of rapid and weak pulse, narrowing of pulse pressure or hypotension, and the presence of cold clammy skin; and DHF grade IV is profound shock with undetectable blood pressure and pulse [4]. Grades III and IV are grouped as dengue shock syndrome (DSS) [4]. The definitive diagnosis of dengue illness is made by positive result(s) of serology testing [5], and these serology tests are unfortunately not always readily available at most clinical laboratories. As a result, the clinically mild-form dengue has been inevitably underreported [6, 7], and it is uncommon that clinicians fail to make a timely detection of the early stage of a dengue before it evolves into an overt clinical severe-form DHF. Clinicians inexperienced with dengue may not be alert to this infection entity, and this is especially true for clinicians in a nondengue endemic setting. Once DSS developed, the mortality rate in the affected patients might soar to as high as 20% [8]. It is not uncommon that dengue outbreaks are recognized only when hundreds of people are affected [7], making containment of dengue epidemics difficult and challenging. The importance of a timely diagnosis of dengue illness cannot be overemphasized.

Dengue epidemic was once absent in Taiwan after 1942 [9, 10]. It was not until the 1980s that a number of dengue epidemics reemerged, and of them, two remarkably large ones occurred in 1988 and 2002 in the southern part of this island [9, 10]. The rest were sporadic dengue clusters, and there was a small number of silent dengue transmissions between some of these dengue clusters [11, 12]. Owing to the absence of large-scale dengue epidemics like those annually found in southeastern Asian countries [2], most clinicians in Taiwan are not experienced with dengue illness.

In 2002 a dengue epidemic due to dengue virus serotype 2 (DENV-2) developed in southern Taiwan in which more than 5000 symptomatic cases were found and most of the affected patients were adults [10, 13], and thanks to the convenience of medical access in Taiwan, a large number of febrile patients presented to Emergency Services of Kaohsiung Chang Gung Memorial Hospital (KSCGMH) seeking medical help because of their concern for possible dengue illness. KSCGMH is a 2500-bed medical facility serving as a primary care and tertiary referral centre in this area. Complying with the law, clinicians notified Center for Disease Control (CDC, Taiwan) of patients with suspected DF/DHF and sampled patients' blood specimens for the central laboratory of CDC for serologic confirmation of dengue. Of note, blood specimens of a substantial number of suspicious dengue patients were also subject to clinical-practice-based laboratory investigations/tests such as bacterial culture, complete blood count (CBC), coagulation tests, and blood chemistry analysis, as the diagnoses of dengue were doubtful and further testing to exclude other diseases was therefore needed. To elucidate whether the clinical-practice-based laboratory data play a complementary role in facilitating the diagnosis of DF/DHF, the aims of this study were to estimate the sensitivity, specificity, accuracy, positive predictive value (PPV), and negative predictive value (NPV) of the individual data and varied combinations of each of these data retrieved from hemogram (i.e., peripheral white cell count [WBC], platelet count [PC]), coagulation profile (i.e., prothrombin time [PT], activated partial thromboplastin time [APTT]), and blood chemistry (i.e., alanine aminotransferase [ALT] and aspartate aminotransferase [AST]) in the diagnosis of DF/DHF. The above-mentioned data were selected for analyses, because with the exception of PT, they were frequently found to be abnormal in dengue-affected patients [10, 13–17]. The results of this study provide valuable information that potentially helps build up a justified suspicious index for DF/DHF and/or facilitates the diagnosis of DF/DHF before the results of serological tests for dengue are available.

## 2. Patients and Methods

Potentially eligible included patients who were adults aged ≧18 years with a tentative clinical diagnosis of DF/DHF treated at Emergency Services or during admission at KSCGMH between July and November 2002. After excluding those with missing data, a total of 200 patients were eventually included for analyses. Half of the included patients who were subsequently proven to be serologically dengue-positive were allocated as the overall dengue cases, while another half who were serologically dengue-negative were allocated as the nondengue cases. A definitive diagnosis of dengue was made when at least one of the following serologic test results was found: (i) a positive reverse-transcriptase polymerase detection of DENV, (ii) a positive enzyme-linked immunosorbent assay result for specific immunoglobulin M antibody for DENV in acute-phase serum, and (iii) a fourfold or higher increase in dengue-specific hemagglutination inhibition titers in convalescent serum as compared to that in acute-phase serum [13–16]. The serologic tests for DENV infection were performed by CDC, Taiwan. Patients of the overall dengue cases were separated into those with DF (DF cases) and those with DHF (DHF cases) for further analyses [4].

A retrospective chart review was carried out for collection of demographic, clinical, and clinical-practice-based laboratory information of the included patients. The retrieved WBC, PC, PT, APTT, ALT, and AST were assayed from the specimens sampled from the affected patients when the tentative diagnosis of DF/DHF was made. Each of these data was regarded as an individual variable. Each individual variable and varied combined variables of the overall dengue cases, DF cases, and DHF cases were separately compared to the corresponding ones of nondengue cases. Individual components in combined variables were results of assays of blood specimens sampled on the same day. Because a large number of variables were derived from combination of individual variables and because each of these combined variables were regarded as one individual variable in analysis, to make the complexity more legible, combined variable A and variable B were expressed as a variable A + B and so on. The Chi-squared test or Fisher exact test was used for comparison of dichotomous variables, while the Student's *t*-test or Mann-Whitney *U* test was used for comparison of continuous variables. A 2-tailed *P* was considered statistically significant.

To assess the values of the aforementioned data in facilitating the suspicion/diagnoses of dengue in general and DF/DHF in particular, we calculated the sensitivity, specificity, accuracy, PPV, and NPV based on these individual laboratory variables and varied combinations of each of them [18]. Because of the trade-off between the measurements of sensitivity and specificity, and because of variables with high accuracy suggesting the coexistence of a potentially acceptable sensitivity and specificity, individual laboratory variables and combined variables with a calculated accuracy >0.80 in the diagnosis and DF and/or DHF would be further examined with the receiver-operating-characteristic (ROC) curve analysis plotting sensitivity against 1-specificity [19, 20]. The area under the curve (AUC) with its 95% confidence interval of each separately constructed ROC curve was measured using SPSS 15 software for Windows (SPSS Inc., Chicago, Ill) to obtain the predictive accuracy of these clinical-practice-based laboratory data in the diagnosis of DF and/or DHF. AUC between 0.90 and 1 was considered excellent, between 0.80 and 0.90 good, between 0.70 and 0.80 fair, between 0.60 and 0.70 poor, and between 0.50 and 0.60 fail [20].

## 3. Results 

### 3.1. Demographic, Clinical, and Laboratory Information of Patients in Dengue and Nondengue Cases

The overall dengue case and nondengue case groups each included 100 patients, and the former was made up of 57 DF cases and 43 DHF cases. Similar demographics but a number of significant differences in clinical manifestations (Table 1) and clinical-practice-based laboratory data (Table 2) were found when the overall dengue cases, DF cases, and DHF cases were separately compared with the nondengue cases.

### 3.2. Sensitivity, Specificity, PPV, NPV, and Accuracy of Laboratory Data in the Diagnoses of the Overall Dengue, DF, and DHF

The sensitivity, specificity, PPV, NPV and accuracy calculated from individual laboratory variables and varied combinations of them in the diagnosis of DF and/or DHF are summarized in Table 3.

In the diagnosis of overall dengue, variables with a high sensitivity, in decreasing order, were PC < 150 × 10^9^ cells/L (100%), PC < 100 × 10^9^ cells/L (97%), prolonged APTT (89.7%), prolonged APTT + PC < 150 × 10^9^ cells/L (89.6%), prolonged APTT + normal PT (89.4%), prolonged APTT + normal PT + PC < 150 × 10^9^ cells/L (89.4%), prolonged APTT + PC < 100 × 10^9^ cells/L (88.6%), and prolonged APTT + normal PT + PC < 100 × 10^9^ cells/L (87.9%). The specificity of PC < 150 × 10^9^ cells/L and PC < 100 × 10^9^ cells/L was 48.8% and 70%, respectively. Prolonged APTT + normal PT + PC < 100 × 10^9^ cells/L had an accuracy of 84.6% (sensitivity 87.9%, specificity 78.9%, PPV 87.9%, and NPV 78.9%), while PC < 100 × 10^9^ cells/L had an accuracy of 83.5% (sensitivity 97.0%, specificity 70.0%, PPV 76.4%, and NPV 95.9%).

In the diagnosis of DF, variables with a high sensitivity, in decreasing order, were PC < 150 × 10^9^ cells/L (100%), PC < 100 × 10^9^ cells/L (94.7%), prolonged APTT + normal PT + PC < 150 × 10^9^ cells/L (91.7%), prolonged APTT (91.7%), prolonged APTT + normal PT (91.7%), prolonged APTT + PC < 100 × 10^9^ cells/L (88.9%), and prolonged APTT + normal PT + PC < 100 × 10^9^ cells/L (88.9%). Among them, prolonged APTT + normal PT + PC < 100 × 10^9^ cells/L had an accuracy of 83.8% (specificity 78.9%, PPV 80%, and NPV 88.2%). The specificity of PC < 150 × 10^9^ cells/L and PC < 100 × 10^9^ cells/L was 29.0% and 70%, respectively.

In the diagnosis of DHF, variables with high sensitivity, in decreasing order, were PC < 150 × 10^9^ cells/L (100%), PC < 100 × 10^9^ cells/L (100%), AST > 40 U/L (97.1%), PC < 150 × 10^9^ cells/L + AST > 40 U/L (97.1%), PC < 100 × 10^9^ cells/L + AST > 40 U/L (97.1%), ALT > 40 U/L + AST > 40 U/L (92.6%), PC < 150 × 10^9^ cells/L + ALT > 40 U/L + AST > 40 U/L (92.6%), and PC < 100 × 10^9^ cells/L + ALT > 40 U/L + AST > 40 U/L (92.6%). The specificity of PC < 150 × 10^9^ cells/L and PC<100 × 10^9^ cells/L was 29% and 70%, respectively. Variables with high accuracy (>80.0%) were PC < 100 × 10^9^ cells/L + ALT > 40 U/L + AST > 40 U/L (sensitivity 92.6%, specificity 76.6%, PPV 69.4%, and NPV 94.7%), prolonged APTT + normal PT + PC < 100 × 10^9^ cells/L (sensitivity 86.7%, specificity 78.9%, PPV 76.5%, and NPV 88.2%), prolonged APTT + normal PT + AST > 40 U/L (sensitivity 88.5%, specificity 75%, PPV 76.7%, and NPV 87.5%), prolonged APTT + normal PT + ALT > 40 U/L (sensitivity 81%, specificity 81.5%, PPV 77.3%, and NPV 84.6%), and PC < 100 × 10^9^ cells/L + ALT > 40 U/L (sensitivity 89.3%, specificity 75.5%, PPV 65.8%, and NPV 93%).

### 3.3. Sensitivity, Specificity, PPV, NPV, Accuracy, and AUC of Laboratory Variables Included in ROC Curve Analysis

PC < 100 × 10^9^ cells/L, prolonged APTT + normal PT + PC < 100 × 10^9^ cells/L, PC < 100 × 10^9^ cells/L + ALT > 40 U/L, PC < 100 × 10^9^ cells/L + ALT > 40 U/L + AST > 40 U/L, prolonged APTT + normal PT + AST > 40 U/L, and prolonged APTT + normal PT + ALT > 40 U/L were included for ROC analysis. AUC, along with the sensitivity, specificity, PPV, NPV, and accuracy in the diagnosis of DF and/or DHF is summarized in Table 4. PC < 100 × 10^9^ cells/L and prolonged APTT + normal PT + PC < 100 × 10^9^ cells/L each had a good predictive accuracy (AUC > 0.8) in the diagnoses of the overall dengue, DF, and DHF, while the remaining variables had a good predictive accuracy only in the diagnosis of DHF.

## 4. Discussion

The immunopathogenesis of DF/DHF is characterized by an aberrant immune overactivation and cytokine overproduction that lead to the development of a great array of clinical and laboratory manifestations [17, 21–23]. Some of the cytokines are proinflammatory, while others are anti-inflammatory [17, 21–23]. These cytokines are capable of causing leukocytes to activate synergistically or antagonistically [17, 24], and clinical and laboratory manifestations in the dengue affected patients are the net effect of the interactions between one another among these activated cytokines [17, 21–23].

Myelosuppression in DF/DHF leads to leukopenia [24], and some of the dengue-affected patients experience prior transient neutrophilia and monocytosis before development of leukopenia [24]. DEV-2 was reported to be able to bind to human platelets in the presence of virus-specific antibodies [25]. As a result of molecular mimicry, autoantibodies produced in DF/DHF patients are capable of coating human platelets [17], and IFN-*γ* activates macrophages to phagocytosize the auto antibody-coated platelets, rendering thrombocytopenia [24]. During acute DENV infection, both coagulation and fibrinolysis are activated, leading to alterations in coagulation parameters (e.g., platelet count and APTT) and fibrinolytic parameters (e.g., tissue-type plasminogen [tPA] and plasminogen activator inhibitor [tAPI]). APTT prolongs as tPA increases. The activations of coagulation and fibrinolysis are much more drastic in DHF/DSS than in DF [24, 26].

An APTT prolongation and normal PT often found in DHF suggest a defect in the intrinsic pathway of coagulation, which is caused by either downregulation of the synthesis or overconsumption of specific factor(s) that are presumably produced by hepatocytes [24]. Hepatitis is usually found in the acute phase of DF/DHF [24, 27]. Data derived from the analysis of the linear correlation and regression between the levels of AST/ALT and APTT show a strong association between them, suggesting that hepatic dysfunction might be responsible for the decreased synthesis of specific factors in the coagulation intrinsic pathway [24, 28]. Increased factor consumption as indicated by the high levels of tPA is also associated with APTT prolongation [26]. Elevated liver enzymes are especially found in patients with DHF [27], and this may explain the findings that variables made up of PC < 100 × 10^9^ cells/L and/or prolonged APTT + normal PT with an elevated AST and/or an elevated ALT had a good accuracy in the diagnosis of DHF but not in the diagnosis of the overall dengue or DF.

Given significant differences in clinical manifestations between the overall dengue/DF/DHF cases and the nondengue cases in this series and in others [29], clinicians experienced with these infectious disease entities may not often have difficulty making the diagnosis of DF/DHF on clinical basis, especially in areas where DF/DHF is always endemic [29]. On the other hand, for inexperienced clinicians the clinical diagnosis of DF/DHF is often a big challenge. The scenarios in which clinical-based suspicion/diagnosis of DF/DHF is challenging include inexperienced clinicians' facing febrile patients in a small dengue cluster or encountering febrile travelers from dengue-endemic locales to non-dengue-endemic area. The significant differences in the daily-practice-based laboratory data between patients with DF/DHF and the nondengue cases (Table 2) suggested these individual data alone and/or in combination with other(s) potentially facilitate the suspicion/diagnosis of DF/DHF. One study from Singapore where dengue was found all year round reported that a model combining clinical feature (skin rash) and laboratory parameters (white cell count, hemoglobin, PT, creatinine, and bilirubin levels) was able to distinguish dengue illness (mainly DF) from other infections with a sensitivity of 84% and specificity of 85% [29].

While ROC plots provide a global comprehensive view of the test, sensitivity and specificity describe the test's ability to correctly distinguish between DF/DHF and nondengue patients [19]. As PC < 100 × 10^9^ cells/L had a high sensitivity but low specificity in the diagnosis of DF and/or DHF, it may be useful in screening dengue illness when this viral infection is rarely encountered. Prolonged APTT + normal PT + PC < 100 × 10^9^ cells/L with high sensitivity (87.9% for the overall dengue, 88.9% for DF, and 86.7% for DHF) and a comparatively high specificity of 78.9% for DF and/or DHF in this series suggest that the combined variable is especially useful in screening dengue illness during a dengue epidemic or in countries where dengue is always endemic, as under these circumstances, it is likely that clinicians tend to make a tentative diagnosis of dengue in most febrile patients lacking obvious localizing signs to suggest an alternative diagnosis [29, 30].

In the diagnosis of DHF, APTT + normal PT + PC < 100 × 10^9^ cells/L, PC < 100 × 10^9^ cells/L + ALT > 40 U/L, PC < 100 × 10^9^ cells/L + ALT > 40 U/L + AST > 40 U/L, prolonged APTT + normal PT + AST > 40 U/L, and prolonged APTT + normal PT + ALT > 40 U/L were found to have a comparable sensitivity and specificity in the diagnosis of DHF. However, when facing a patient with an underlying liver dysfunction due to viral hepatitis and/or fatty liver, APTT + normal PT + PC < 100 × 10^9^ cells/L is the variable of choice for screening DHF.

Predictive value, a calculation of the percentage of correct negative or correct positive result, is applicable once the prevalence of a disease is taken into consideration [19]. The potential roles played by individual variable/varied combined variables in the diagnoses of the overall dengue/DF/DHF are summarized in Table 4. While trade-off between sensitivity and specificity, and/or suboptimal PPV/NPV was found at measurements using other variable/varied combined variables, prolonged APTT + normal PT + PC < 100 × 10^9^ cells/L had a favorable sensitivity, specificity, PPV, and NPV in diagnosis of DF and/or DHF. To make the applicability of these clinical-practice-based laboratory data simplified and user-friendly, we propose prolonged APTT + normal PT + PC < 100 × 10^9^ cells/L be used for screening and evaluating the likelihood of DF and/or DHF.

The present study implies that daily-practice-based laboratory data play a complementary role in prompting the suspicion and/or facilitating the diagnosis of DF and/or DHF, which is especially important for clinicians who are inexperienced with these infectious entities. In addition to providing an appropriate therapeutic guidance, a timely suspicion and diagnosis of dengue infection may help the public health authorities launch necessary containment measures earlier, thus diminishing the amplitude of a dengue epidemic that would otherwise be a much larger one.

As DF/DHF features dynamically changing clinical and laboratory manifestations within a few days [14, 16, 31, 32], clinicians may repeatedly sample serum specimens for daily-practice-based laboratory tests if the initial ones do not disclose clear enough information for evaluation of the likelihood of DF/DHF.

Our data were obtained from adult patients during a dengue epidemic due to DENV-2 in Taiwan. Of our serologically dengue-negative patients, 78% suffered viral infections other than dengue and 6% suffered bacterial sepsis, while the rest 22% experienced miscellaneous diseases (see footnote of Table 1 for details). The entities of febrile illness in the nondengue patients might affect the measurements of sensitivity, specificity, PPV, and NPV for dengue illness using the clinical-practice-based laboratory data in our study, and this is one of the limitations that deserves attention. As clinical and laboratory manifestations in DF/DHF result from sophisticated immunologic reactions [17, 24, 33], which may vary from patients in one series to another depending on the genetics of the hosts and the culprit viruses [34, 35], additional limitations of our study must be addressed. It is uncertain whether these daily-practice-based data are applicable in facilitating diagnosis of DF/DHF in adults of other race and/or DF/DHF caused by DENV of other serotypes. Likewise, it is uncertain whether these daily-practice data are applicable in facilitating diagnosis of DF/DHF in pediatric patients. Further study is merited to clarify these important questions, as the answers potentially greatly impact medicine practice in dengue epidemics which are distributed worldwide, mainly in tropical areas where medical resources are deficient.

## Figures and Tables

**Table 1:** Demographic and clinical information of the included patients.

Variable	Overall dengue cases (A) (*N* = 100)	*P* (A versus D)	Dengue fever cases (B) (*N* = 57)	*P* (B versus D)	Dengue hemorrhagic cases (C) (*N* = 43)	*P* (C versus D)	Nondengue cases^1^ (D) (*N* = 100)
Demographics							
Age, yr		0.375		0.669		0.279	
Mean (±SD)	46.1 ± 11.5		45.1 ± 12.3		41.2 ± 10.5		44.9 ± 17.6
Median (range)	49 (18–68)		47 (18–68)		49 (18–63)		43 (18–81)
Male gender, no. (%)	44 (44)	0.667	22 (38.6)	>0.99	22 (51.2)	0.270	40 (40)
Underlying condition,^2^ no. (%)							
Hypertension	13 (13)	>0.99	6 (10.5)	0.801	7 (16.3)	0.607	13 (13)
Diabetes mellitus	14 (14)	0.834	5 (8.8)	0.603	9 (20.9)	0.199	12 (12)
Old stroke	0	0.121	0	0.297	0	0.316	4 (4)
Chronic kidney disease	0	0.121	0	0.297	0	0.316	4 (4)
Solid tumor	0	0.246	0	0.554	0	0.554	3 (3)
Symptom/sign,^3^ no. (%)							
Fever	96 (96)	0.251	57 (100)	0.027	39 (90.7)	>0.99	91 (91)
Bone pain	55 (55)	0.007	33 (57.9)	0.007	22 (51.2)	0.093	35 (35)
Retroorbital pain	8 (8)	>0.99	6 (10.5)	0.782	2 (4.7)	0.505	9 (9)
Arthralgia	10 (10)	0.033	6 (10.5)	0.027	4 (9.3)	0.067	2 (2)
Abdominal pain	40 (40)	0.1	20 (35.1)	0.373	20 (46.5)	0.036	28 (28)
Cough	31 (31)	0.342	16 (28.1)	0.574	15 (34.9)	0.220	24 (24)
Diarrhea	22 (22)	0.197	11 (19.3)	0.497	11 (25.6)	0.148	14 (14)
Nausea/vomiting	36 (36)	0.042	19 (33.3)	0.134	17 (39.5)	0.041	22 (22)
Rash	34 (34)	0.005	24 (42.1)	0.001	10 (23.3)	0.347	16 (16)
Myalgia	15 (15)	<0.001	10 (17.5)	<0.001	5 (11.6)	<0.001	47 (47)
Petechiae	44 (44)	<0.001	19 (33.3)	<0.001	25 (58.1)	<0.001	9 (9)
Gum bleeding	26 (26)	<0.001	13 (22.8)	<0.001	13 (30.2)	<0.001	0
Gastrointestinal bleeding	20 (20)	<0.001	7 (12.3)	0.001	13 (30.2)	<0.001	0

^1^Including viral infections other than dengue illness (78 cases), bacterial sepsis (6 cases), gastrointestinal bleeding with/without liver cirrhosis (4 cases), urinary tract infection (2 cases), and aplastic anemia, colitis, acute hepatitis, acute pancreatitis, hepatocellular carcinoma, biliary tract infection, aseptic meningitis, systemic lupus erythematosus, acute tonsillitis, and pneumonia (each 1 case).

^2^An individual patient might have more than one underlying condition.

^3^An individual patient might have more than one symptom/sign.

**Table 2:** Laboratory data of the included patients.

Variable	Overall dengue cases (A) (*N* = 100)	*P* (A versus D)	Dengue fever cases (B) (*N* = 57)	*P* (B versus D)	Dengue hemorrhagic cases (C) (*N* = 43)	*P* (C versus D)	Nondengue cases^1^ (D) (*N* = 100)
Leukopenia (WBC < 3.0 × 10^9^ cells/L), no. (%)	49 (40)	0.001	31 (54.4)	<0.001	18 (41.9)	0.049	25 (25)
Platelet count < 150.0 (×10^9^ cells/L), no. (%)	100 (100)	<0.001	57 (100)	<0.001	43 (100)	<0.001	71 (71)
Platelet count < 100.0 (×10^9^ cells/L), no. (%)	97 (97)	<0.001	54 (94.7)	<0.001	43 (100)	<0.001	30 (30)
Prolonged APTT,^2^ *n*/*N* (%)	61/68 (89.7)	0.001	33/36 (91.7)	0.006	28/32 (87.5)	0.030	26/41 (63.4)
Prolonged PT,^3^ *n*/*N* (%)	1/68 (1.5)	0.023	0/37	0.055	1/31 (3.2)	0.217	5/39 (12.8)
AST > 40 U/L (normal value, < 40 U/L), *n*/*N* (%)	68/79 (86.1)	<0.001	34/44 (77.3)	<0.001	34/35 (97.1)	<0.001	27/64 (42.2)
ALT > 40 U/L (normal value, < 40 U/L), *n*/*N* (%)	49/65 (75.4)	<0.001	24/37 (64.9)	0.006	25/28 (89.3)	<0.001	19/55 (34.5)

Abbreviations: APTT: activated partial thromboplastin time; PT: prothrombin time; AST: aspartate aminotransferase; ALT: alanine aminotransferase; *n*/*N*: no. of patients/no. of patients with data available.

^1^See footnote in Table 1 for details.

^2^Prolonged APTT was defined as an increased APTT value > 20% of the control value.

^3^Prolonged PT was defined as an increased PT value > 3 seconds than that of control.

**Table 3:** Sensitivity, specificity, positive predictive value, negative predictive value, and accuracy of laboratory data in the diagnoses of overall dengue, DF, and DHF^1^.

Variable^2^	Overall dengue cases, *n*/*N* (%)	DF cases, *n*/*N* (%)	DHF cases, *n*/*N* (%)	Nondengue cases, *n*/*N* ^3^ (%)
Prolonged APTT	61/68 (89.7)^4^	33/36 (91.7)^4,7^	28/32 (87.5)^4^	26/41 (63.4)
Leukopenia	49/100 (49.0)	31/57 (54.4)	18/43 (41.9)	25/100 (25.0)
Platelet < 150 × 10^9^ cells/L	100/100 (100)^4,7^	57/57 (100)^4,7^	43/43 (100)^4,7^	71/100 (71.0)
ALT > 40 U/L	49/65 (75.4)	24/37 (64.9)	25/28 (89.3)^4,7^	19/55 (34.5)
AST > 40 U/L	68/79 (86.0)^4^	34/44 (77.3)	34/35 (97)^4,7^	27/64 (42.3)
Prolonged APTT + leukopenia	32/68 (47.1)	18/36 (50.0)	14/32 (43.8)	14/41 (34.1)
Prolonged APTT + platelet < 150 × 10^9^ cells/L	60/67 (89.6)^4^	32/35 (91.4)^4^	28/32 (87.5)^4,7^	21/41 (51.2)
Prolonged APTT + ALT > 40 U/L	35/50 (70.0)	18/28 (64.3)	17/22 (77.3)^7^	9/30 (30.0)
Prolonged APTT + AST > 40 U/L	50/60 (83.3)^4,6^	26/32 (81.3)^4^	24/28 (85.7)^4,7^	12/31 (38.7)
Prolonged APTT + leukopenia + platelet < 150 × 10^9^ cells/L	32/68 (47.0)	18/36 (50.0)	14/32 (43.8)	12/41 (29.3)
Prolonged APTT + leukopenia + ALT > 40 U/L	18/50 (36.0)^5^	8/28 (28.6)^5^	10/22 (45.5)^5^	5/30 (16.7)
Prolonged APTT + leukopenia + AST > 40 U/L	27/60 (45.0)^5,6^	15/32 (46.9)^5^	12/28 (42.9)^5^	5/31 (16.1)
Prolonged APTT + leukopenia + platelet < 150 × 10^9^ cells/L + ALT > 40 U/L	18/50 (36.0)^5^	8/28 (28.6)^5^	10/22 (45.6)^5^	5/30 (16.7)
Prolonged APTT + leukopenia + platelet < 150 × 10^9^ cells/L + AST > 40 U/L	27/60 (45.0)^5,6^	15/32 (46.9)^5^	12/28 (42.9)^5^	5/31 (16.1)
Prolonged APTT + leukopenia + platelet < 150 × 10^9^cells/L + ALT > 40 U/L + AST > 40 U/L	15/52 (28.8)^5^	5/31 (16.1)^5^	10/21 (47.6)^5^	4/29 (13.8)
Leukopenia + platelet < 150 × 10^9^ cells/L	49/100 (49.0)	31/57 (54.4)	18/43 (41.9)	23/100 (23.0)
Leukopenia + ALT > 40 U/L	22/65 (33.8)^5^	11/37 (29.7)^5^	11/28 (39.3)^5^	7/55 (12.7)
Leukopenia + AST > 40 U/L	32/79 (40.5)^5^	18/44 (40.9)^5^	14/35 (40.0)^5^	9/64 (14.1)
Leukopenia + platelet < 150 × 10^9^ cells/L + ALT > 40 U/L	22/65 (33.8)^5^	11/37 (29.7)^5^	11/28 (39.3)^5^	7/55 (12.7)
Leukopenia + platelet < 150 × 10^9^ cells/L + AST > 40 U/L	32/78 (41.0)^5^	18/44 (40.9)^5^	14/34 (41.2)^5^	9/64 (14.1)
Leukopenia + platelet < 150 × 10^9^ cells/L + ALT > 40 U/L + AST > 40 U/L	21/60 (35.0)^5^	10/33 (30.3)^5^	11/27 (40.7)^5^	6/47 (12.8)
Platelet < 150 × 10^9^ cells/L + ALT > 40 U/L	49/65 (75.4)	24/37 (64.9)	25/28 (89.3)^4,7^	17/55 (30.9)
Platelet < 150 × 10^9^ cells/L + AST > 40 U/L	67/78 (85.9)^4^	33/43 (76.7)	34/35 (97.1)^4,7^	25/64 (39.1)
Platelet < 150 × 10^9^cells/L + ALT > 40 U/L + AST > 40 U/L	46/59 (77.9)	21/32 (65.6)	25/27 (92.6)^4,7^	13/47 (27.7)
ALT > 40 U/L + AST > 40 U/L	48/60 (80.0)	23/33 (69.7)	25/27 (92.6)^4,7^	14/47 (29.8)
Prolonged APTT + normal PT	59/66 (89.4)^4^	33/36 (91.7)^4,7^	26/30 (86.7)^4,7^	19/38 (50.0)
Prolonged APTT + normal PT + leukopenia	30/66 (45.6)	18/36 (50)	12/30 (40.0)	12/38 (31.6)
Prolonged APTT + normal PT + platelet < 150 × 10^9^ cells/L	59/66 (89.4)^4,6^	33/36 (91.7)^4,7^	26/30 (86.7)^4,7^	14/38 (36.8)
**Prolonged APTT + normal PT + ALT > 40 U/L**	35/49 (71.4)^5,6^	18/28 (64.3)^5^	17/21 (80.9)^4,5,8^	5/27 (18.5)
**Prolonged APTT + normal PT + ALT > 40 U/L**	49/58 (84.5)^4,6,8^	26/32 (81.3)^4^	23/26 (88.5)^4,7,8^	7/28 (25.0)
Prolonged APTT + normal PT + leukopenia + platelet < 150 × 10^9^ cells/L	30/66 (45.5)	18/36 (50)	12/30 (40)	10/38 (26.3)
Prolonged APTT + normal PT + leukopenia + ALT > 40 U/L	18/49 (36.7)^5,6^	8/28 (28.6)^5^	10/21 (47.6)^5^	3/27 (11.1)
Prolonged APTT + normal PT + leukopenia + AST > 40 U/L	26/58 (44.8)^5,6^	15/32 (46.9)^5^	11/26 (42.3)^5^	4/28 (14.3)
Prolonged APTT + normal PT + leukopenia + platelet < 150 × 10^9^cells/L + ALT > 40 U/L	18/49 (36.7)^5,6^	8/28 (28.6)^5^	10/21 (47.6)^5^	3/27 (11.1)
Prolonged APTT + normal PT + leukopenia + platelet < 150 × 10^9^cells/L + AST > 40 U/L	26/57 (45.6)^5,6^	15/32 (46.9)^5^	11/25 (44.0)^5^	4/28 (14.3)
Prolonged APTT + normal PT + leukopenia + platelet < 150 × 10^9^cells/L + ALT > 40 U/L + AST > 40 U/L	18/46 (39.1)^5,6^	8/26 (30.8)^5^	10/20 (50.0)^5^	3/26 (11.5)
**Platelet < **100 × 10^9^ ** cells/L**	97/100 (97)^4,7,8^	54/57 (94.7)^4,7^	43/43 (100)^4,7^	30/100 (30.0)
Prolonged APTT + platelet < 100 × 10^9^ cells/L	60/68 (88.2)^4,6^	32/36 (88.9)^4,7^	28/32 (87.5)^4,7^	14/41 (34.1)
Prolonged APTT + leukopenia + platelet < 100 × 10^9^ cells/L	31/68 (45.6)^5^	17/36 (47.2)^5^	14/32 (43.8)^5^	8/41 (19.5)
Prolonged APTT + leukopenia + platelet < 100 × 10^9^cells/L + ALT > 40 U/L	17/50 (34.0)^5^	7/28 (25)^5^	10/22 (45.5)^5^	5/30 (16.7)
Prolonged APTT + leukopenia + platelet < 100 × 10^9^cells/L + AST > 40 U/L	26/60 (43.3)^5,6^	14/32 (43.8)^5^	12/28 (42.9)^5^	5/31 (16.1)
Prolonged APTT + leukopenia + platelet < 100 × 10^9^cells/L + ALT > 40 U/L + AST > 40 U/L	17/47 (36.2)^5,6^	7/26 (26.9)^5^	10/21 (47.6) ^5^	4/29 (13.8)
Leukopenia + platelet < 100×10^9^ cells/L	47/100 (47)^5^	29/57 (50.9)^5^	18/43 (41.9) ^5^	14/100 (14.0)
Leukopenia + platelet < 100 × 10^9^cells/L + ALT > 40 U/L	21/65 (32.3)^5^	10/37 (27)^5^	11/28 (39.3)^5^	7/55 (12.7)
Leukopenia + platelet < 100 × 10^9^ cells/L + AST > 40 U/L	31/78 (39.7)^5^	17/44 (38.6)^5^	14/34 (41.2)^5^	9/64 (14.1)
Leukopenia + platelet < 100 × 10^9^cells/L + ALT > 40 U/L + AST > 40 U/L	20/60 (33.4)^5^	9/33 (27.3)^5^	11/27 (40.7)^5^	6/47 (12.8)
**Platelet < **100 × 10^9^ **cells/L + ALT > 40 U/L**	48/65 (73.8)	23/37 (62.2)	25/28 (89.3)^4,7,8^	13/53 (24.5)
Platelet < 100 × 10^9^cells/L + AST > 40 U/L	67/79 (84.8)^4^	33/44 (75)^7^	34/35 (97.1)^4,7^	19/64 (29.7)
**Platelet < **100 × 10^9^ **cells/L + ALT > 40 U/L + AST > 40 U/L**	46/60 (76.7)^6^	21/33 (63.6)	25/27 (92.6)^4,7,8^	11/47 (23.4)
**Prolonged APTT + normal PT + platelet < **100 × 10^9^ ** cells/L**	58/66 (87.9)^4,6,8^	32/36 (88.9)^4,6,7,8^	26/30 (86.7)^4,7,8^	8/38 (21.1)
Prolonged APTT + normal PT + leukopenia + platelet < 100 × 10^9^ cells/L	29/66 (43.9)^5,6^	17/36 (47.2)^5^	12/30 (40.0)^5^	6/38 (15.8)
Prolonged APTT + normal PT + leukopenia + platelet < 100 × 10^9^ cells/L + ALT > 40 U/L	17/49 (34.7)^5,6^	7/28 (25)^5^	10/21 (47.6)^5^	3/27 (11.1)
Prolonged APTT + normal PT + leukopenia + platelet < 100 × 10^9^cells/L + AST > 40 U/L	25/57 (43.9)^5,6^	14/32 (43.6)^5^	11/25 (44.0)^5^	4/28 (14.3)
Prolonged APTT + normal PT + leukopenia + platelet < 100 × 10^9^cells/L + ALT > 40 U/L + AST > 40 U/L	17/46 (36.9)^5,6^	7/26 (26.9)^5^	10/20 (50.0)^5^	3/26 (11.5)

Abbreviations: APTT: activated partial thromboplastin time; PT: prothrombin time; AST: aspartate aminotransferase; ALT: alanine aminotransferase; *n*/*N*: no. of patients/no. of patients with data available.

^1^Sensitivity = (number of true-positives) × 100/(number of true-positives + number of false-negatives), specificity = (number of true-negatives) × 100/(number of true-negatives + number of false-positives), accuracy = (number of true-positives + number of true-negatives) × 100/(total instances), PPV = (number of true-positives) × 100/(number of true-positives + number of false-positives), and NPV = (number of true-negatives) × 100/(number of true-negatives + number of false-negatives) [18].

^2^Leukopenia was defined as peripheral white cell count < 3.0 × 10^9^ cells/L, prolonged APTT as an increased APTT value > 20% of the control value, and prolonged PT as an increased PT value > 3 seconds than that of control. Variables in bold font are those that had an accuracy > 80% in the diagnosis of the DF and/or DHF and were therefore subjected to the receiver-operating-characteristic (ROC) curve analysis in which the under the curve (AUC) was measured to obtain the predictive accuracy (see Table 4 for details).

^3^See footnote in Table 1 for details.

^4^Variable with a sensitivity > 80%.

^5^Variable with a specificity > 80%.

^6^Variable with a positive predictive value > 80%.

^7^Variable with a negative predictive value > 80%.

^8^Variable with an accuracy > 80%.

**Table 4:** Sensitivity, specificity, PPV, NPV, accuracy, and AUC of laboratory variables included in ROC curve analysis^1^.

Laboratory parameter	Overall dengue						DF						DHF					
	Sn (%)	Sp (%)	PPV (%)	NPV (%)	Ac (%)	AUC (95% CI)	Sn (%)	Sp (%)	PPV (%)	NPV (%)	Ac (%)	AUC (95% CI)	Sn (%)	Sp (%)	PPV (%)	NPV (%)	Ac (%)	AUC (95% CI)
PC < 100 × 10^9^ cells/L	97.0	70.0	76.4	95.9	83.5	**0.835 (0.775–0.895)**	94.7	70.0	64.3	95.9	79.0	**0.824 (0.751–0.890)**	100	70	58.9	100	79.0	**0.850 (0.789–0.911)**
Prolonged APTT + normal PT + PC < 100 × 10^9^ cells/L	87.9	78.9	87.9	78.9	84.6	**0.834 (0.746–0.923)**	88.9	78.9	80.0	88.2	83.8	**0.839 (0.742–0.936)**	86.7	78.9	76.5	88.2	82.4	**0.828 (0.724–0.932)**
PC < 100 × 10^9^ cells/L + ALT > 40 U/L						0.747						0.699	89.3	75.5	65.8	93	80.2	**0.852 (0.761–0.943)**
PC < 100 × 10^9^ cells/L + ALT > 40 U/L + AST > 40 U/L						0.766						0.701	92.6	76.6	69.4	94.7	82.4	**0.846 (0.752–0.939)**
Prolonged APTT + normal PT + AST > 40 U/L						0.791						0.781	88.5	75.0	76.7	87.5	81.5	**0.817 (0.698–0.937)**
Prolonged APTT + normal PT + ALT > 40 U/L						0.765						0.729	81.0	81.5	77.3	84.6	81.3	**0.812 (0.682–0.942)**

Abbreviations: Sn: sensitivity; Sp: specificity; PPV: positive predictive value; NPV: negative predictive value; Ac: accuracy; AUC: area under the receiver-operator characteristic curves; DF: dengue fever; DHF: dengue hemorrhagic fever; CI: confidence interval; PC: platelet count; APTT: activated partial thromboplastin time; PT: prothrombin time; AST: aspartate aminotransferase; ALT: alanine aminotransferase.

^1^Only sensitivity, specificity, positive predictive value, and negative predictive value of variables having an accuracy with an AUC > 0.8 (in bold font) in ROC analysis are shown. Calculations of sensitivity, specificity, positive predictive value, negative predictive value, and accuracy were based on formulae put in the footnote of Table 3.
